# Fabry disease: Definition, Incidence, Clinical presentations and Treatment *-* Focus on cardiac involvement

**DOI:** 10.12669/pjms.38.8.7063

**Published:** 2022

**Authors:** Sahrai Saeed, Massimo Imazio

**Affiliations:** 1 Sahrai Saeed, Department of Heart Disease, Haukeland University Hospital, Bergen, Norway; 2 Massimo Imazio, Cardiology, Cardiothoracic Department, University Hospital “Santa Maria della Misericordia”, ASUFC, Udine, Italy.

**Keywords:** Fabry disease, Cardiomyopathy, Echocardiography, Left ventricular hypertrophy, Enzyme replacement therapy

## Abstract

Fabry disease (FD) is a relatively rare X-linked hereditary disease caused by mutations in the GLA gene that results in deficient α-galactosidase A (α-Gal A) enzyme activity. The disturbed catabolism of the neutral sphingolipids globotriaosylceramide (Gb3) leads to its progressive lysosomal accumulation throughout the body. Multiple organs can be affected. The atypical late-onset cardiac variant is associated with a high burden of cardiac morbidity and mortality. The aim of this work was to present an updated overview of the FD, with focus on cardiovascular manifestations and its management. Enzyme replacement therapy (ERT) is nowadays an established treatment of FD and is recommended as early as possible with or without chaperone therapy (migalastat) to prevent or delay the progression of renal, cardiac, and cerebrovascular complications. It improves quality of life and may further result in decrease in Left ventricular (LV) mass and to some extent LV function recovery. However, LV hypertrophy (LVH) does not always respond well to ERT despite successful Gb3 clearance. Furthermore, its impact on the hard clinical events is uncertain. Some possible reasons for this apparent discrepancy are discussed. ERT may be less effective in patients who have already developed fibrosis or irreversible organ damage. However, other confounding factors may be equally important.

## INTRODUCTION

### Definition and incidence:

Fabry disease (FD), also known as Anderson-Fabry disease, was initially described independently by Johannes Fabry in Germany and William Anderson in England in 1898.^1^,^2^ It is a rare X-linked hereditary disease caused by mutations in the GLA gene encoding the lysosomal enzyme alpha-galactosidase A (α-Gal A). The deficient α-Gal A activity leads to lysosomal deposition of the glycosphingolipids globotriaosylceramide (Gb3) and its derivatives in different cells of the body particularly in the microvascular endothelial, vascular smooth muscle, renal tubular cells and podocytes, neuronal cells and cardiomyocytes.^3^-^5^ The incidence of FD was reported to range from one in 40000 to one in 117000 live male births.^6^ This estimate may be low because screening in newborns suggested a much higher prevalence of up to one in 8800 newborns.^7^ Targeted screening programs assessing individuals on dialysis and those with hypertrophic cardiomyopathies (HCM) suggests that the atypical later-onset FD, which primarily affects the cardiovascular, cerebrovascular and renal system is more common than previously assumed.^8^,^9^

### Clinical presentations:

FD is a progressive disease that affects multiple organs with a wide range of clinical manifestations. The heart, brain/central nervous system and kidneys are most commonly affected in the late-onset forms (Table-I). Premature death is often attributed to progressive heart failure, arrhythmia, stroke, and renal failure.^10^ There are different phenotypes of FD. The classic early-onset form occurs in males with <1% α-Gal A enzyme activity in childhood or adolescence, characterized by periodic crises of severe pain in the extremities (acroparesthesias), appearance of vascular cutaneous lesions (angiokeratomas), sweating abnormalities, corneal and lenticular opacities (seen in 80-90% of carriers), and proteinuria.^11^ The late-onset, milder form occurring in males (sixth to eight decade) with greater than 1% α-Gal A activity, is also referred to as atypical variants, i.e. cardiac variant. Disease severity may vary in females from rarely being asymptomatic to have more severe symptoms as observed in males with the classic phenotype. Heterozygous females have enzymatic activity levels of α-Gal A ranging from 0−12.6 μmol/h/L in dried blood spots and have typically milder symptoms at a later age of onset than males. It is believed that compared with general population, patients with FD have a reduced life expectancy by approximately 20 years in males and by approximately 15 years in females.^12^,^13^

**Table-I:** Organ-specific manifestations in Fabry disease.

***Early signs and symptoms of classical Fabry disease in childhood and adolescence***
Peripheral neuropathy
Periodic crises of severe pain in the extremities (acroparesthesia)
Neuro-otological (hearing abnormalities)
Opthalmic (corneal verticillata, vasculopathy)
Skin: Angiokeratomas and sweating abnormalities (anhidrosis, hypohidrosis or hyperhidrosis)
Gastrointestinal symptoms (nausea, vomiting, diarrhoea, abdominal pain, weight gain difficulty)
***Progressive renal dysfunction leading to end-stage renal disease and need for dialysis (men in 3^rd^-5^th^ decade)***
Microalbuminuria/proteinuria
Hyperfiltration
Fibrosis, sclerosis, tubular atrophy
***Cardiovascular manifestations***
LVH (regional, apical, global)
Increased endomyocardial trabeculation
LV apical aneurysm
LV dysfunction, reduced strain (global and regional/segmental)
RV hypertrophy
Reduced RV free wall strain
Immune-mediate myocarditis
Myocardial fibrosis
Congestive heart failure
Increased filling pressure, diastolic dysfunction
Aortic and mitral valve thickening (deposition of Gb3), prolapse or regurgitation
LV outflow tract obstruction
Reduced LA function (reservoir strain and strain rate)
Mild aortic root dilatation
Coronary microvascular dysfunction
Arrhythmias
Beat to beat variation in heart rate
Bradycardia
Chronotropic incompetence
Atrioventricular blocks
Short PR-interval, LV strain and LVH on ECG
Elevated cardiac biomarkers (NT-proBNP, high-sensitivity troponins)
Increased arterial stiffness
***Cerebrovascular manifestations***
Stroke/TIA
Vascular abnormalities (vertebrobasilar dolichoectasia and vessel tortuosity)
Epilepsy
Neuropsychiatric (depression & cognitive decline)
***Neuroradiological/MRI findings in Fabry disease***
White matter hyperintensities
Infarcts (lacunar and cortical/territorial)
Cerebral microbleeds
Intracerebral haemorrhage
Pulvinar sign^*37^
***Pulmonary manifestations (may mimic chronic obstructive pulmonary disease)^38^***
Wheezing, dyspnoea, dry cough

* A highly specific sign of FD seen on T1 weighted images (often in males), associated with cardiomyopathy and end-stage renal disease, and may be due to calcification related to blood flow changes.^37^LV, left ventricular; LVH, left ventricular hypertrophy; RV, right ventricular.

In addition to cardiac involvement (discussed later), cerebrovascular manifestations are also common in FD. Data from Fabry outcome survey (FOS) has suggested that stroke or TIA occur in approximately 13% (15% males versus 11.5% females) of all patients with FD including those with classic Fabry and late-onset cerebrovascular disease.^11^,^14^ Overall, large lacunar infarcts are described in about 20% of patients with FD. Large cortical “territorial” infarcts are less common. MRI findings in FD are valuable with regard to diagnosis, understanding the mechanisms of disease and prognosis. Conventional MRI findings include white matter hyperintensities (WMHs), infarcts (lacunar and cortical), cerebral microbleeds (CMBs), intracerebral haemorrhage (Table-I). WMHs are the most commonly reported MRI findings in FD, which increase with age.^15^

FD is also a known cause of stroke in young patients, but the prevalence is probably underestimated. In the European Stroke in Young Fabry Patients Study (SIFAP) study of 5023 stroke patients (age 18-55 years),^16^ FD was a cause of stroke in 1% of cases compared with a prevalence of 0.2% in older unselected stroke patients.^17^ However, the true prevalence FD in stroke patients remains unclear as there are limited data available.

Finally, with regard to coronavirus disease 2019 (COVID-19), the current view is that pre-existent organ damage and inflammation may predispose patients with more advanced FD to a more severe course of COVID-19. By contrast, patients with less advanced FD do not appear to be more susceptible than the general population.^18^

### Diagnosis:

The most reliable method for diagnosing FD in males is identification of deficient α-Gal A enzyme activity in plasma, isolated leukocytes, and/or cultured cells, which can be further confirmed by identification of a hemizygous pathogenic variant in GLA by genetic testing.^10^-^13^,^19^ Of note, males with classic FD have <1% α-Gal A enzyme activity and those with atypical FD have >1% α-Gal A enzyme activity.^11^ In female probands, identification of a heterozygous GLA pathogenic variant by molecular genetic testing is required as some females may have normal α-Gal A enzyme activity. Plasma lyso-Gb3 is a useful biomarker with good correlation with disease severity and treatment response (clinical efficacy of ERT) and can be used to differentiate the various clinical phenotypes as well as understand the mutation.

Finally, in a report from Norway, two unrelated families with cardiomyopathy due to lysosomal accumulation of Gb3 mimicking Fabry disease were also described.^20^ All patients had global left ventricular hypertrophy (LVH), normal α-gal A activity and no mutations in the Fabry gene. Molecular genetic analysis is important and can differentiate the various phenotypes such as well-known typical exogenic Fabry mutations and late onset pathogenic mutations from harmless mutations, unclear polymorphism, and unknown new mutations. It is important to note that absence of a known family history does not preclude the diagnosis because *de novo* or spontaneous mutations have been documented.

### Fabry cardiomyopathy:

Cardiac involvement in FD is quite frequent and is the main cause of death in patients with advanced disease. Fabry cardiomyopathy is typically characterized by a progressive LVH, leading to myocardial fibrosis, LV systolic dysfunction, ischemia and arrhythmias. A detailed list of cardiovascular manifestation in FD and potential complications are presented in Table-I.

Commonly reported electrocardiographic (ECG) findings include bradycardia and PQ-interval shortening, which has been shown to be due to shortening of the P-wave duration and one of the first signs of cardiac involvement. Of note, increasing age has been demonstrated to be associated with PQ- and QRS-interval prolongation and left QRS axis deviation as well as a progressive sinus and AV node disease, necessitating a close monitoring for bradyarrhythmias and the implantation of a pacemaker. Accordingly, prolongation of the PQ interval is a very common finding in the natural history of FD patients, more likely reflecting a progressively increasing disease burden and age-related degenerative processes. In FD, an abnormal ECG at the time of treatment initiation was significantly associated with cardiac disease progression independent of age, gender and LV mass at baseline.^21^

**Fig.1:**
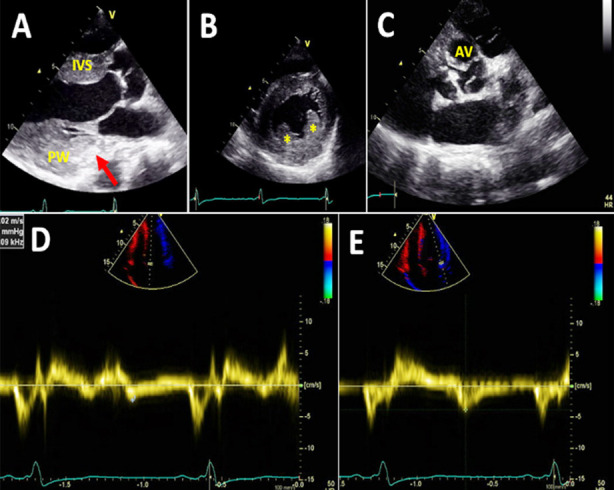
Echocardiographic images of a male Fabry patient in his 50s. ***A*** is a parasternal long-axis view showing increased thickness of interventricular septum (IVS) and posterior wall (PW) and hyperechogenic signal in the basal part of posterior wall. ***B*** is a parasternal short-axis view and illustrates left ventricular hypertrophy with prominent papillary muscles (asterisk). Please note the posterior wall is thicker than the interventricular septum despite correct alignment. ***C*** is another parasternal short-axis view demonstrating a thickened tricuspid aortic valve (AV). ***D*** and ***E*** are tissue Doppler images, displaying very low septal (D) and lateral (E) annular velocities.

Echocardiography is often the first-line imaging modality for the evaluation of cardiac damage in FD, particularly to assess LV function and LVH (Fig.1). Novel techniques such as 2D and 3D speckle-tracking with strain images provide valuable insights into subclinical LV impairment that would not be possible to detect with conventional echocardiography (ejection fraction). LVH in FD may morphological and clinically resemble sarcomere-gene-associated HCM, and genetic testing is often needed to establish the diagnosis.^22^ Particularly, in patients with late-onset cardiac variant of FD with unexplained LVH, the differentiation from other HCMs may be difficult, if no other systemic manifestations are present - because various types of LVH have been reported in FD.^23^,^24^ By systematic screenings for GLA mutations in HCM patients without sarcomere gene mutations, GLA mutations were found in 3% of HCM families and in 10% of females without sarcomere-gene mutations.^25^ Other studies of large cohorts of HCM patients have identified FD as the cause of LVH in 0.5% to 1% of cases.^26^,^27^ Screening of males with “late-onset” HCM found that 6.3% who were diagnosed at 40 years of age or later, and 1.4% of males who were diagnosed before age 40 years had proven FD by identification of low α-Gal A enzyme activity and a genetic testing (GLA hemizygous pathogenic variant).^28^

Adverse cardiac outcomes in FD are associated with age, disease severity and advanced cardiac disease but not with the presence of cardiac genetic variants.^29^ Age has a strong correlation with the severity of LVH. In a study of 55 female patients (age range 6.1-70.8 years) with proven FD, Kampmann et al. showed that all patient older than 45 years had LVH.^30^ However, in female Fabry patients, a decline in LV function and development of fibrosis do not necessarily require LVH. In a study of 104 patients (58 females, age 42±16 years and 46 males, age 42±13 years) with genetically proven FD, late gadolinium enhancement (LGE) on cardiac magnetic resonance (CMR) was never observed in men with LV wall thickness <12 mm, but in female patients, LGE was already detectable with an LV wall thickness of 9 mm.^31^ Of note, LGE was detected in 23% of the female patients without LVH, but was never seen in male patients with normal LV mass.

**Fig.2:**
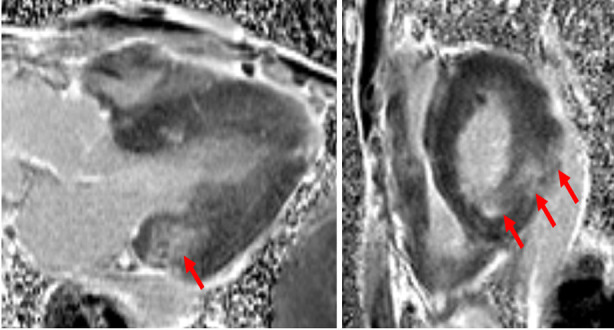
Cardiac magnetic resonance images of the same patient as in Fig.1. Left panel is vertical 3-chamber view and right panel parasternal short axis view demonstrating areas of LGE in the basal posterior and inferolateral walls (arrows). LGE, late gadolinium enhancement.

It is known that male patients with Fabry cardiomyopathy more often exhibit progressive myocardial fibrosis (Fig.2). Search for fibrosis on imaging modalities is important for identifying individuals in high risk for organ damage and cardiovascular complications. Also, knowledge of the presence or absence of myocardial fibrosis is crucial before the start of ERT because this affects the level of expectations.^32^ LGE on CMR, reflecting replacement fibrosis, is of important prognostic value and detect subclinical LV dysfunction, even in female patients with apparently normal LV mass and wall thickness. About 50% of patients display fibrosis as assessed by LGE on CMR.^33^ The distribution of LGE may differ within the myocardium. In patients with LVH, the LGE may be mid-myocardial, while transmural LGE with LV-thinning can also occur. Fibrosis was initially thought to be caused by tissue ischemia secondary to endothelial accumulation of glycosphingolipids in the microvasculature. However, in addition to the neutral glycosphingolipid (Gb3) accumulation, other mechanisms such as inflammation and immune system activation, which subsequently lead to irreversible organ damage, are also involved in cardiac damage in FD.^23^ An additional value of CMR is to provide a more accurate tissue characterization by non-contrast myocardial T1 mapping. T1 mapping is highly sensitive for tissue characterization and differential diagnoses of Fabry cardiomyopathy. T1 mapping demonstrates typically low mean septal T1 in FD that indicates fat accumulation in the myocardium suggesting glycosphingolipid accumulation in cardiomyocytes at earlier stages. This is useful in differentiating LVH due to FD from other common aetiologies such as hypertensive heart disease and amyloidosis in which fibrosis is expected to be associated with T1 prolongation. T1 may increase with the developing of myocardial fibrosis at late stages of the disease. Of note, even in the absence of LVH or fibrosis, the T1 may still be lower than normal in FD, indicating an early myocardial involvement. This is particularly useful in asymptomatic women without LVH (phenotype-negative carriers of pathogenic α-galactosidase gene mutations).

On histopathology in patient with Fabry cardiomyopathy, extensive myocyte vacuolation and collagenous fibrosis have been observed.^34^ Furthermore, autopsy studies have shown biventricular hypertrophy, particularly LVH with localized thinning at the base of the LV posterior wall.^35^ Right ventricle (RV) can be also involved. Most recently, it was shown that patients with Fabry cardiomyopathy had impaired RV mechanics characterized by more pronounced impairment of RV free-wall strain in comparison with sarcomeric hypertrophic cardiomyopathy.^36^

Myocardial ischaemia and angina can also occur in patients with FD and may be due to functional remodelling of the coronary arteries, microvascular dysfunction,^39^ endothelial dysfunction and oxygen demand-supply mismatch of the hypertrophic myocardium.^40^ However, inflammation and activation of the immune system have also been proposed as alternative mechanisms. New studies indicate that immune-mediated myocarditis is common in FD and can be revealed by antiheart/antimyosin autoantibodies, or in the advanced stage by a CMR.^41^ The proinflammatory response of the myocardium occurs with the disease progression and is believed to be generated by the glycosphingolipid accumulation in the cardiomyocytes. This may limit the impact of ERT and require concomitant implemental immunosuppressive therapy.^42^

Assessment of myocardial deformation by strain and strain rate analysis has been shown to be a sensitive method to detect subclinical LV dysfunction (when conventional EF is still normal) and early myocardial damage in FD compared with healthy age-and gender-matched controls, independent of the presence of LVH.^43^ This is particularly important in carriers of pathogenic variants. Conventional diagnostic methods such as ECG and 2D-echo may not be useful to assess pre-clinical LV dysfunction. Interestingly, in majority of patients with FD and normal EF without LVH, segmental strain was reduced in the basal-posterior and lateral walls.^44^ Lower longitudinal strain in the fibrotic wall segments has also been shown to be associated with LGE. Therefore, global longitudinal strain derived from speckle-tracking echocardiography can be a good functional marker and useful tool for the indirect evaluation of LGE in Fabry cardiomyopathy.^45^

In addition to LV remodelling, left atrial (LA) remodelling is also evident in FD. It is believed that LA enlargement and reduced atrial compliance occur before the development of LVH.^46^ Although patients with HCM had larger LA volumes, LA function (reservoir strain and strain rate) was severely decreased in both conditions.^47^

**Fig.3:**
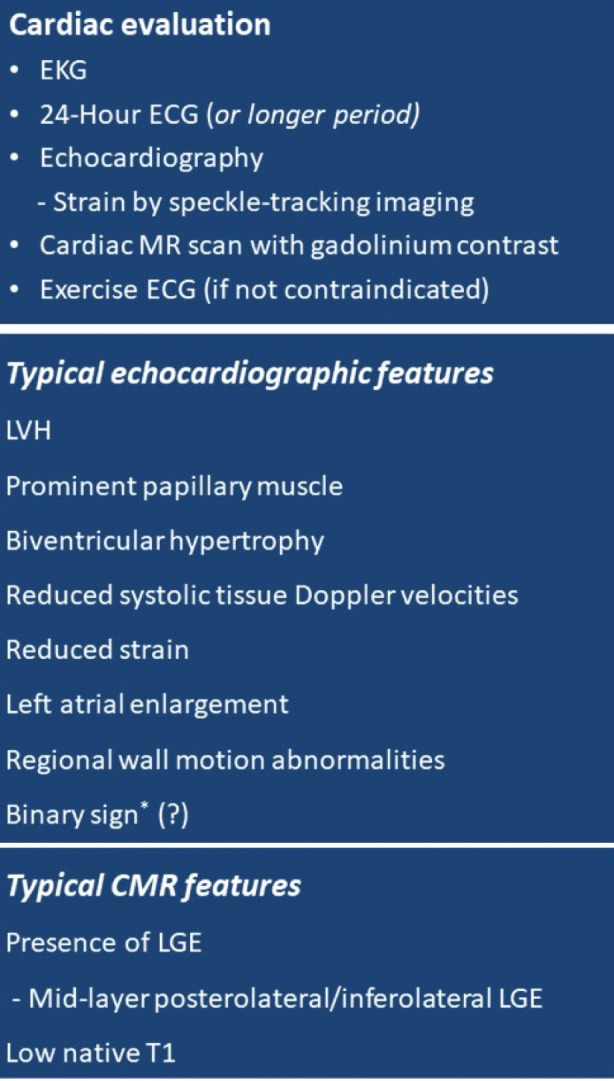
A simplified guide for cardiac assessment and common echocardiographic and cardiac magnetic resonance (CMR) features pathognomonic of Fabry disease. LVH, left ventricular hypertrophy; LGE, late gadolinium enhancement. ^*^Binary appearance of left ventricular endocardial border, may suggest endomyocardial Gb3 accumulation (48).

The current guidance and expert opinion recommend cardiac assessment including EKG and echocardiography to be performed annually in males from 18 years of age and biannually in women from age 18 to 35 years.^11^ CMR is valuable both at baseline (low native T1) and during follow up to monitor disease staging and treatment response. A simplified guidance on common CMR and echocardiographic features which are pathognomonic for Fabry cardiomyopathy is presented in Fig.3.^48^ High-sensitivity troponins are important for staging of the cardiomyopathy.

### Therapeutic options, focus on ERT:

**Fig.4:**
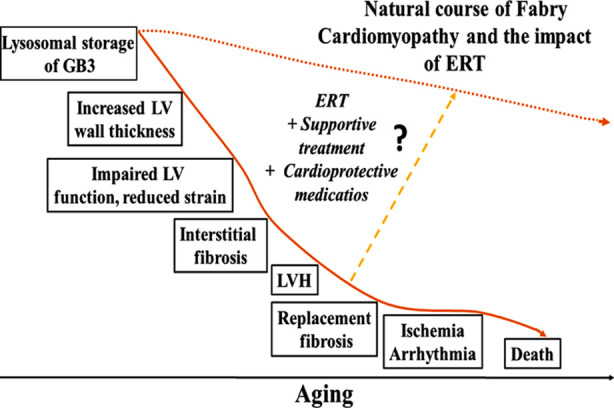
A simplified model showing the Natural course of Fabry Cardiomyopathy and the possible impact of enzyme replacement therapy (ERT). LVH, left ventricular hypertrophy.

Before the introduction of enzyme replacement therapy (ERT) 21 years ago, treatment of FD was mainly supportive/symptom-based and included medications for neuropathic pain and acroparesthesia, aspirin, statin, antihypertensive and cardioprotective drugs (ACE inhibitors or angiotensin receptor blockers to reduce proteinuria, improve cardiac and kidney function, reduce LV mass and blood pressure, and improve endothelial function.^49^), betablockers, antianginal (nitroglycerine and calcium channel blockers), antiarrhythmic drugs, ICD/pacemaker, anticoagulation, and haemodialysis and renal transplantation for patients with end-stage renal disease. ERT is now the cornerstone of FD management and is recommended with or without chaperone therapy (migalastat) to prevent or delay the progression of renal, cardiac, and cerebrovascular complications. The currently two available ERTs: agalsidase alfa (Replagal) and agalsidase beta (Fabrazyme) both have shown their efficacy in clinical studies regarding clearance of Gb3 from plasma, kidney cells and cardiomyocytes.^50^-^52^ There are no differences in the clinical efficacy of agalsidase alfa or agalsidase beta.^53^ Both products could improve quality of life, reduce, or stabilize LV mass, preserve renal function, and slow down the decline in glomerular function.^51^,^54^-^57^ These findings seem promising and ERT is nowadays an established treatment for patients with FD and recommended to be started as early as possible to prevent or delay the progression of organ damage and modify the natural course of FD (Fig.4). Generally, in patients with FD and cardiac and renal involvement, an early start of ERT is superior to the late start in order to achieve better long-term outcome.^56^ There is also evidence about arterial stiffness reduction following ERT. Collin *et al*. demonstrated a successful reduction in aortic stiffness assessed by carotid-femoral pulse wave velocity, and LVH following long-term ERT in patients with FD.^58^ Regarding the impact on reverse LV remodelling, previous studies have documented LV mass regression on ERT in patients with FD, particularly when combined with optimal antihypertensive treatment; however, its impact on the progression of Fabry cardiomyopathy and expected treatment outcomes, particularly regarding major cardiovascular adverse events have been questionable.^59^-^63^ A Danish Fabry cohort study failed to show that ERT significantly altered the progression of cardiac involvement compared to patients not receiving ERT.^64^ Indeed, ERT group showed progression of symptoms and had more concomitant cardiac medications prescribed. This raised concerns in terms of the efficacy and clinical benefit of ERT for patients with cardiac involvement in FD. There are no RCTs evaluating the effects of ERT (considered unethical) and most evidence come from observational retrospective cohort studies. However, some factors related to ERT; i.e., short circulatory half-life, dose and the induction of antibodies towards the recombinant proteins may be responsible for the limited treatment efficacy.^65^ In addition to irreversible organ damage *per se*, whether age, gender and other factors such as immune-mediated myocarditis and pro-inflammatory pathways, or other unknown confounders dilute the effect of ERT is subject of future research, especially focusing on a more personalised approach.

## CONCLUSIONS

FD is a serious life-threatening disease which can affect multiple organs of the body. The atypical late-onset cardiac variant is associated with a high burden of cardiovascular morbidity and mortality. Accurate and timely diagnosis of FD offers the possibility of disease-specific treatment with sustained clinical benefits. All the available evidence suggest that in classical FD, early intervention offers the best chance of reducing morbidity and improving life expectancy. Disease-specific treatments have shown promising results. Specifically, ERT has impacted the clinical course of FD and life expectancy, when it is started early enough, ideally before the occurrence of target organ damage. However, the effects of disease-specific treatment can be limited in patients with late-onset cardiac variant with a diagnostic delay, presenting with more severe organ damage. Hence, it is important to avoid misdiagnosis and underdiagnosis of FD, which increases the risk of cardiovascular complications and premature death. Patients with FD need a close follow-up by a multidisciplinary competence team.

Finally, from a clinical point of view an important message from this work can be as follows: In the presence of unexplained LVH, stroke in the young (<50 years) and a decline in kidney function of unknown aetiology, FD should be suspected both in men and women.

### Author Contributions:

**SS** wrote the first draft of the article which was revised by **MI**. Both authors approved the final submission.

### Note:

The opinion expressed in the present expert commentary is the view of the authors and does not necessarily reflect the view of the institutions the authors belong to.
